# Synergistic effects of oxygen vacancies and mesoporous structures in amorphous C@TiO_2_ for photocatalytic CO_2_ reduction

**DOI:** 10.1016/j.isci.2024.110377

**Published:** 2024-06-25

**Authors:** Binxia Yuan, Yuhao Liu, Hong Qian, Rui Zhu, Chengxi Zhang, Weiling Luan

**Affiliations:** 1 College of Energy and Mechanical Engineering, Shanghai University of Electric Power, Shanghai 201306, P.R. China; 2 School of Mechanical and Power Engineering, East China University of Science and Technology, Shanghai 200237, China; 3 Department of Optoelectronic Information Science and Engineering, Jiangsu University of Science and Technology, Zhenjiang 212100, China

**Keywords:** Catalysis, Materials chemistry

## Abstract

In this study, the theoretical calculations proves that the combination of oxygen vacancy and amorphous carbon films in TiO_2_ (V_O_-CT) can effectively reduce the energy bandgap and work function. The minimum Gibbs free energies required for the CO_2_RR reaction of V_O_-CT are 0.20 eV, which is lower than pure TiO_2_. The amorphous c@TiO_2_ nanomaterials with oxygen vacancy and mesoporous structures (V_O_-MCT) are prepared with the P123 surfactant as the template and oxalic acid as an inducer. The electron paramagnetic resonance indicates the presence of abundant oxygen vacancy defects in the samples. UV-vis spectra indicate that the mesoporous structure enhances light absorption capacity. The photocatalytic CO_2_ reduction tests show that the highest conversion rates for CH_4_ and CO of V_O_-MCT are 14 μmol g^−1^ h^−1^ and 10.66 μmol g^−1^ h^−1^, respectively. The electron consumption rate of V_O_-MCT is 12.43 times higher than that of commercial TiO_2_ (P200).

## Highlights

• The synergistic mechanism of mesoporous structure and oxygen vacancy is proposed
• The C@ TiO_2_ with oxygen vacancy and mesoporous structures (V_O_-MCT) are prepared
• The mesoporous structure provides a larger specific surface area of 511 m^2^/g
• The yields for CH_4_ and CO in V_O_-MCT are 14 and 10.66 μmol g^−1^ h^−1^, respectively

## Graphical abstract

**Figure:**
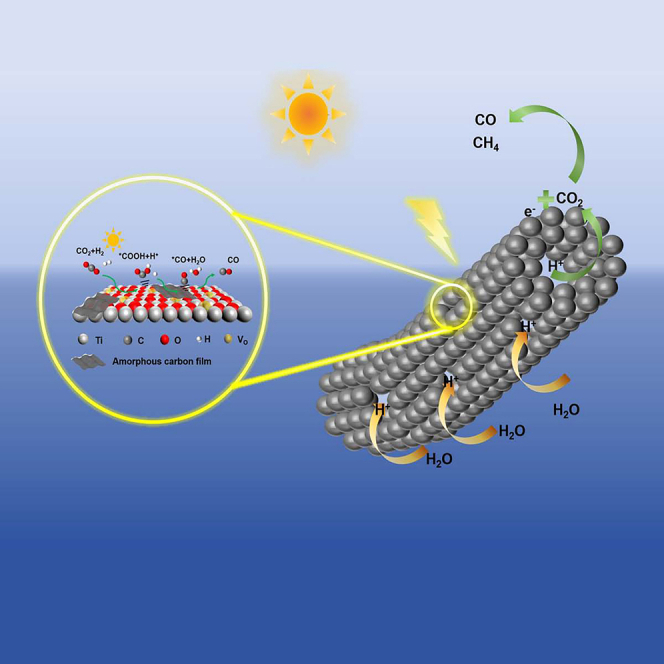

## Introduction

Utilizing sunlight to photocatalytically reduce CO_2_ into C-based fuel has emerged as one of the most promising approaches to address the crises of greenhouse gas emissions and energy shortage.^1^ For instance, the resulting CO can be employed for the industrial production of synthesis gas, while CH_4_ can be utilized in the synthesis of natural gas.^2^^,^^3^ It not only provides a solution to the environmental crisis but also tackles the issue of energy scarcity.

TiO_2_ is one of the most extensively investigated due to its significant potential and challenges in the field of photocatalytic CO_2_ reduction.^4^ However, the wide bandgap restricts its photoabsorption and photocatalytic efficiency under visible light irradiation.^5^ Defect engineering in TiO_2_ is a reliable approach to overcome these challenges. Various methods are employed in TiO_2_ defect engineering, including element doping,^6^ intrinsic oxygen vacancies,^7^ and stress manipulation.^8^ Common dopants, such as Au, Pt, and Ag, significantly increase costs while enhancing light absorption.^9^^,^^10^ Introducing oxygen vacancies on the TiO_2_ surface can provide additional active sites and surface reaction centers, promoting the separation of photogenerated electron-hole pairs and improving photocatalytic activity.^11^^,^^12^^,^^13^ Meantime, the micro-structure of TiO_2_ is crucial in the photocatalytic CO_2_ reduction reaction. The mesoporous structure with a larger specific surface area means more contact sites, leading to higher conversion efficiency.^14^^,^^15^^,^^16^ Although the above research has improved the light absorption ability and photocatalytic performance of TiO_2_ through mesopores or oxygen vacancies,^17^ the overall photocatalytic activity is still relatively low, with average yields of CH_4_ and CO do not exceed 10 μmol g^−1^ h^−1^. Thus, more research is needed to focus on TiO_2_ with both oxygen vacancies and mesoporous structures.

In the article, the aims is to explore the synergistic effects of oxygen vacancies and mesoporous structures in TiO_2_. Through the density functional theory (DFT), the energy band, density of states (DOS), work function, and free energy for the CO_2_ reduction of C coated TiO_2_ with oxygen vacancy are calculated. Meantime, C-coated TiO_2_ with mesoporous structure and oxygen vacancy are synthesized through the sol-gel method and solid-state sintering. By XPS, electron paramagnetic resonance (EPR), BET, SEM, and TEM confirm the presence of amorphous C, oxygen vacancies, and mesoporous structures. The synergistic effect of oxygen vacancies and mesoporous structure in C-TiO_2_ is confirmed through photocatalytic CO_2_ reduction. This study provides ideas for the synergistic improvement of efficient photocatalytic performance by inducing macroscopic mesoporous structures and microscopic defects.

## Results and discussion

### Theoretical calculation analysis

**Figure 1:**
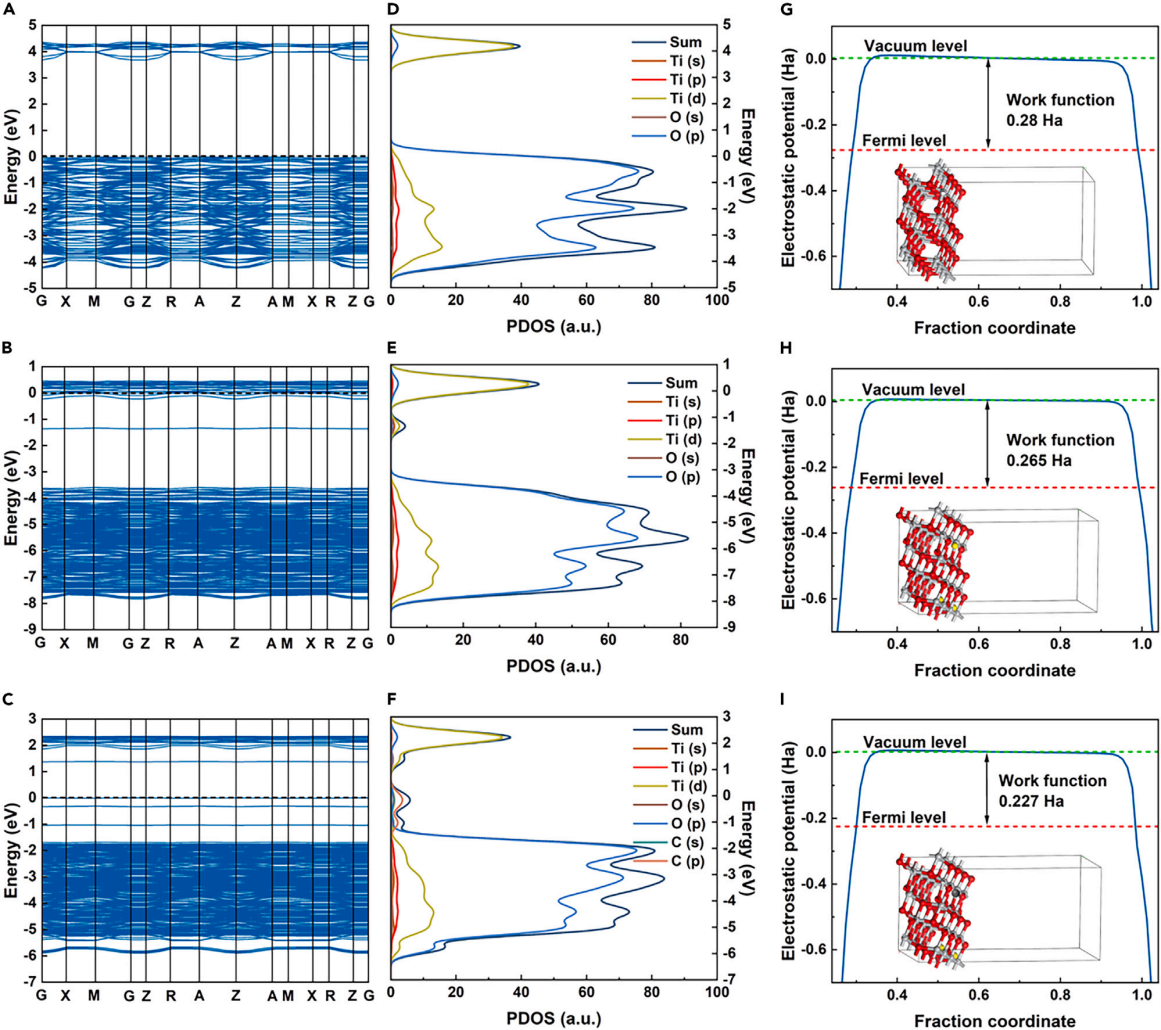
Simulation calculation of energy bands and their electronic properties (A–C) Band structure of (A)T, (B) V_O_-T, (C) V_O_-CT. (D–F) PDOS of (D) T, (E) V_O_-T, (F) V_O_-CT. (G–I) Work function of (G) T, (H) V_O_-T, (I) V_O_-CT. See also Figures S1 and S2.

The effect of oxygen vacancies and C on TiO_2_ is simulated by DFT calculation, as shown in Figure 1. For V_O_-T, a new mid-gap state of Ti (d) orbitals appears in the band gap (Figures 1B and 1E), indicating the formation of defect energy levels due to the presence of oxygen vacancies. In the band structure diagram of V_O_-CT, three intermediate energy gap have been added. The defect energy level in V_O_-CT is not only attributed by the Ti (d) orbital, but also by the C (p) orbital above the valence band (VB). From the PDOS diagram, it can be seen that the density of states distribution of V_O_-CT is more continuous, and the defect intensity is significantly larger than that of V_O_-T. These defective energy levels may facilitate the electronic excitation and e−/h+ separation in TiO_2_, which is important for its photocatalytic and electronic properties.^18^ The band gaps calculated for T, V_O_-T, and V_O_-CT are 3.684 eV, 2.231 eV, and 1.371 eV, respectively. This result demonstrates that the introduction of oxygen vacancies (V_O_) and carbon (C) can modulate the semiconductor band gap. With a smaller band gap, V_O_-CT absorbs less photon energy, enabling the excitation of electrons from the valence band to the conduction band to form electron-hole pairs. The work function of V_O_-CT is 0.227 Ha, which is lower than that of T and V_O_-T models. It indicates C atoms can act as electron channels to promote electron transfer. In addition, the work function of V_O_-T is lower than that of T, indicating that oxygen vacancies are also beneficial for electron transfer. Additionally, the differential charge density maps of the three models depict regions colored in yellow representing gained electrons and regions in blue representing lost electrons, as shown in Figure S2. The yellow range of V_O_-CT surface is greater than that of T and V_O_-T models, indicating a higher propensity for electron gain on the surface of V_O_-CT. The observation is consistent with the results obtained from the work function analysis.

### Structure properties

**Figure 2:**
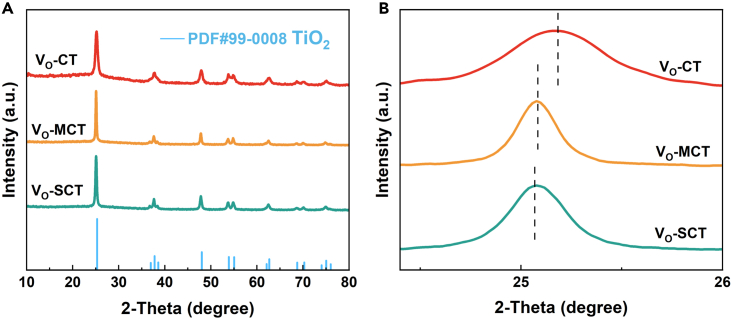
XRD patterns of V_O_-CT, V_O_-MCT, and V_O_-SCT (A) Global graph. (B) (101) crystal planes.

The phase is investigated by XRD analysis (Figure 2). The diffraction peaks of three samples are indexed to the anatase phase of TiO_2_ (JCPDS No.99-0008). There are no obvious diffraction peaks at 31° and 27°, confirming that the synthesized TiO_2_ is a pure anatase phase without the presence of rutile and brookite phases. The diffraction peaks of carbon aren’t observed, which may be due to low carbon content or the presence of amorphous carbon. In Figure 2B, it can be seen that the (101) crystal planes of all samples are shifted to the left, which can be attributed to the presence of oxygen vacancies leading to lattice distortion and a decrease in crystal cell size.^19^ Meanwhile, the offset of V_O_-MCT and V_O_-SCT samples is greater than that of V_O_-CT samples, indicating that they have more lattice distortion.

**Figure 3:**
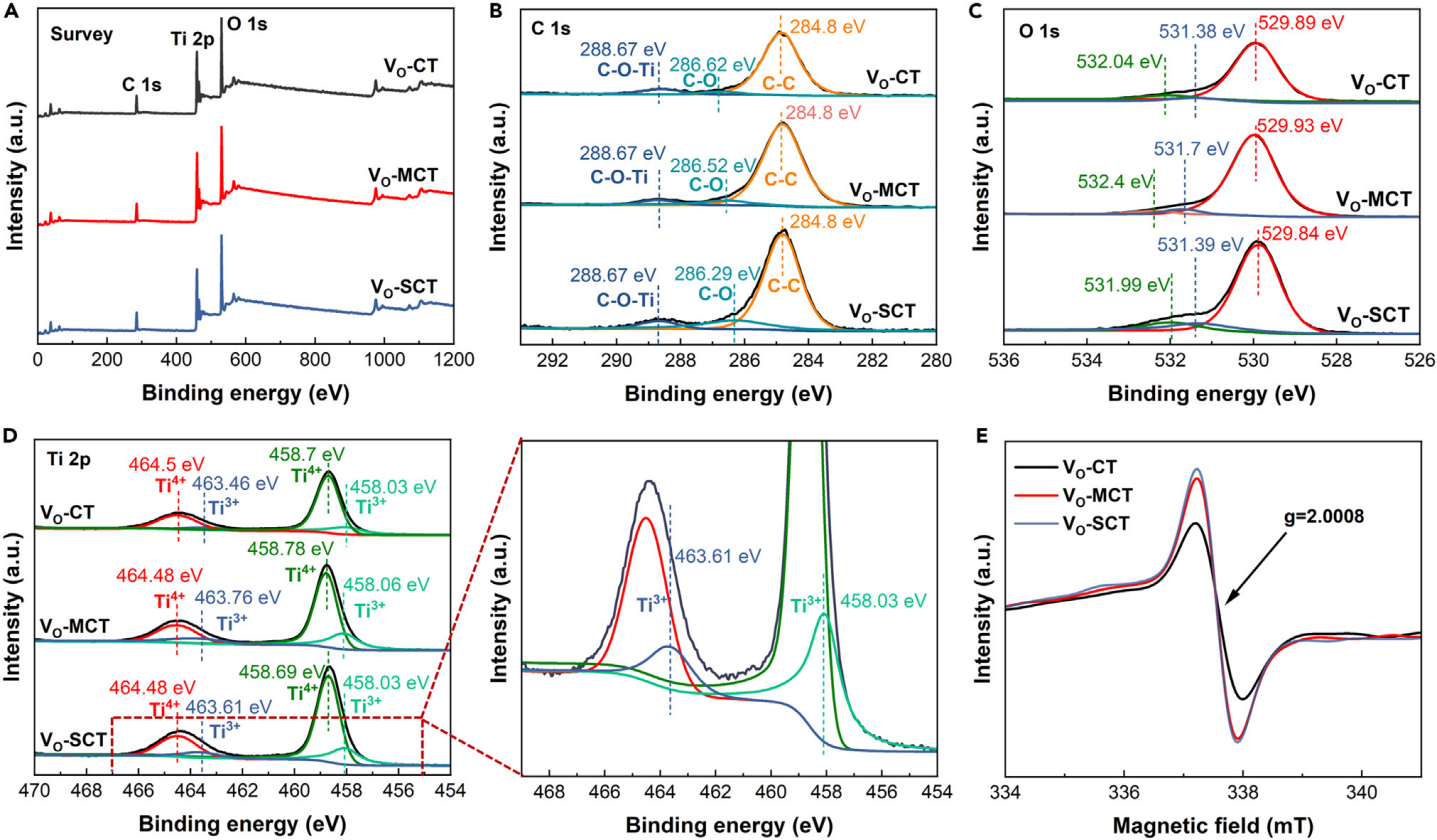
X-ray photoelectron spectroscopy (XPS) and electron paramagnetic resonance spectroscopy (EPR) of different samples (A) Survey of XPS. (B) C1s. (C) O1. (D) Ti 2p. (E) EPR spectra.

XPS analysis is carried out to determine the elemental composition, chemical states, and electronic band structure. The survey spectra confirmed the presence of C, Ti, and O elements in three samples (Figure 3A). In the C1s spectra (Figure 3B), the peak at 284.8 eV is the standard binding energy of the C-C bond. The peak at 288.67 eV can be assigned to the binding energy of C-*O*-Ti species.^20^ The peaks at 286 eV correspond to the binding energies of C-O species,^21^ indicating the presence of C in V_O_-CT, V_O_-MCT, and V_O_-SCT samples. In the O1s spectra (Figure 3C), the prepared samples exhibit a strong peak and two weak peaks. The binding energies observed in V_O_-CT at 532.04 eV and 529.89 eV, in V_O_-MCT at 532.4 eV and 523.93 eV, and in V_O_-SCT at 531.99 eV and 529.84 eV are in close agreement with the standard binding energies of TiO_2_.^22^ The binding energies observed at 531.38 eV, 531.7 eV, and 531.39 eV in the three samples are attributed to the presence of oxygen vacancies.^23^ The formation mechanism of oxygen vacancies is believed to occur through thermodynamic equilibrium. When reaching a certain temperature condition, there is enough energy to enable O atoms to overcome Ti-O chemical bonds, causing them to deviate from their equilibrium positions and form defects.^24^ In the Ti 2p spectra (Figure 3D), the binding energies observed in V_O_-CT at 458.7 eV (Ti 2p_1/2_) and 464.50 eV (Ti 2p_3/2_), in V_O_-MCT at 458.78 eV (Ti 2p_1/2_) and 464.48 eV (Ti 2p_3/2_), and in V_O_-SCT at 458.69 eV (Ti 2p_1/2_) and 464.48 eV (Ti 2p_3/2_) indicate the presence of predominant Ti^4+^ surface states in the samples.^25^ Meantime, the peaks observed in V_O_-CT at 458.03 eV (Ti 2p_1/2_) and 463.46 eV (Ti 2p_3/2_), in V_O_-MCT at 458.06 eV (Ti 2p_1/2_) and 463.76 eV (Ti 2p_3/2_), and in V_O_-SCT at 458.03 eV (Ti 2p_1/2_) and 463.61 eV (Ti 2p_3/2_) are assigned to the Ti^3+^ peaks.^26^ The presence of Ti^3+^ can be attributed to the oxygen vacancies. When there are oxygen vacancies, electrons near Ti^4+^ ions can rearrange and form vacancy induced energy levels. These vacancy induced energy levels allow some electrons to transition from Ti^4+^ ions to vacancy induced energy levels, thereby forming Ti^3+^. Ti^3+^ has partially filled d orbitals, which significantly alter the electronic structure and band structure of the material.^27^

EPR spectra reveal a strong paramagnetic signal peak at g = 2.0008 in all three samples (Figure 3E), indicating the presence of oxygen vacancies (V_O_).^28^ The concentration of V_O_ is directly proportional to the EPR signal intensity, and V_O_-MCT and V_O_-SCT exhibit higher paramagnetic signals than V_O_-CT. This result is consistent with the leftward shift of the TiO_2_ (101) crystal plane diffraction peak in XRD (Figure 2B), confirming that oxygen vacancies cause lattice distortion.

**Figure 4:**
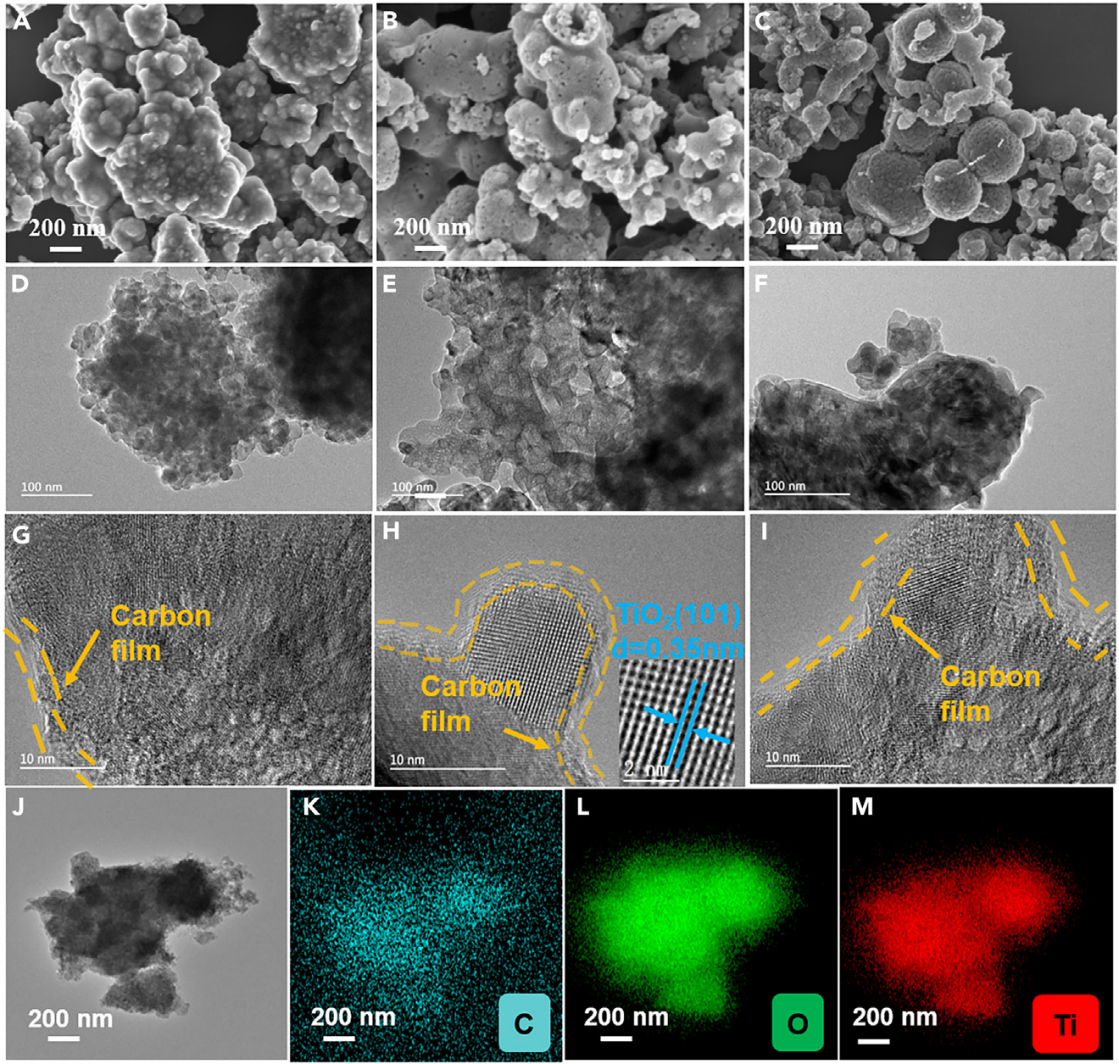
Atomic-resolution structure characterization and EDS patterns of the V_O_-CT, V_O_-MCT, V_O_-SCT (A–C) SEM images of (A) V_O_-CT, (B) V_O_-MCT, (C) V_O_-SCT. (D–F) TEM images of (D) V_O_-CT, (E) V_O_-MCT, (F) V_O_-SCT. (G–I) HRTEM images of (G) V_O_-CT, (H) V_O_-MCT, (I) V_O_-SCT. (J–M) EDS elemental mappings of V_O_-MCT. See also Figure S3.

SEM images revealed the three-dimensional morphology of the prepared samples, as shown in Figures 4A–4C. V_O_-CT is a non porous structure of particle stacking. V_O_-MCT exhits a porous structure with many mesopores and hollow channels on its surface. V_O_-SCT shows spherical aggregates of varying sizes. TEM further confirms the pore free structure in V_O_-CT (Figure 4D). Mesopores are observed in V_O_-MCT, accompanied by numerous hollow structures inside the material (Figure 4E). However, no obvious pore structure is observed in V_O_-SCT (Figure 4F). From the HRTEM image of V_O_-MCT (Figure 4H), lattice fringes with a spacing of 0.35 nm are observed, corresponding to the (101) facet of anatase TiO_2_. Moreover, the HRTEM image of V_O_-MCT reveals the presence of the 2–3 nm amorphous carbon layer at the edges. The stable Ti^3+^ and oxygen vacancies can be attributed to the protective effect of amorphous carbon films on the surface of TiO_2_.^29^ To further elucidate the elemental composition and distribution within V_O_-MCT, elemental mapping analysis (EDS) is conducted. C, O, and Ti signals are detected in the EDS spectrum (Figures 4J–4M). The uniform dispersion of C, O, and Ti atoms confirms the encapsulation of TiO_2_ by amorphous carbon films.^30^ A moderate ratio of oxalic acid and P123 is beneficial for the formation of mesoporous structures, hollow channels, and coral like structures with significant specific surface area. Only an appropriate ratio of oxalic acid and P123 can form a suitable mesoporous structure, see Figure S3.

**Figure 5:**
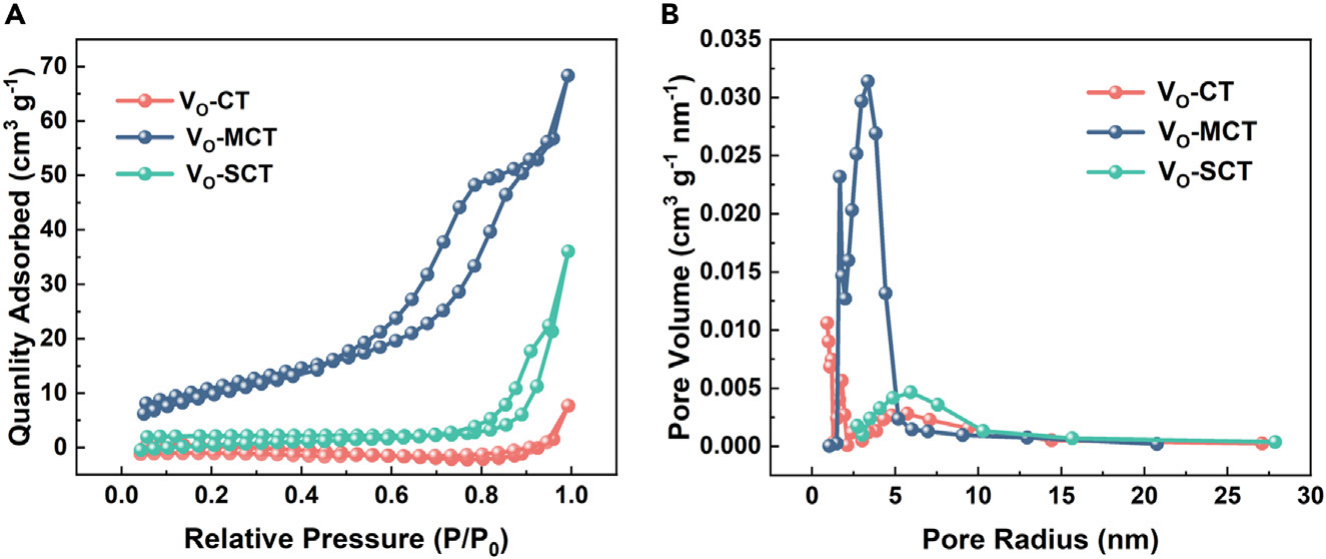
Specific surface area testing (BET) of Vo-T, VO-MCT, and VO-SCT (A) N_2_ adsorption–desorption isotherms. (B) the corresponding BJH pore size distribution of the as-prepared samples.

The N_2_ adsorption-desorption isotherms are employed to characterize the specific surface area and pore volume of the prepared samples (Figure 5). The V_O_-MCT and V_O_-SCT samples exhibit Type IV adsorption isotherms with distinct H3 hysteresis loops. Combined with SEM and TEM images, it is further confirmed that V_O_-MCT has mesoporous properties. The specific surface areas of Vo-T, V_O_-MCT, and V_O_-SCT are calculated to be 12.9405, 511.2061, and 24.9024 m^2^/g, respectively. These results indicate that the quantity of oxalic acid plays a significant role in the formation of mesopores. Oxalic acid is superhydrophilic and TBOT has hydrophilicity, while P123 (EO-PO type) has both hydrophilic and hydrophobic structures. Without the addition of oxalic acid, TBOT and P123 cannot polymerize in hydrophilic hydrophobic structures, resulting in the absence of mesoporous structures after calcination. Excessive oxalic acid leads to the excessive aggregation of TBOT at the hydrophilic end (EO), forming a hydrophobic spherical structure. The above research results demonstrate that amorphous carbon coated mesoporous TiO_2_ composite materials with oxygen vacancy have been successfully prepared.

### Photoelectric performance

**Figure 6:**
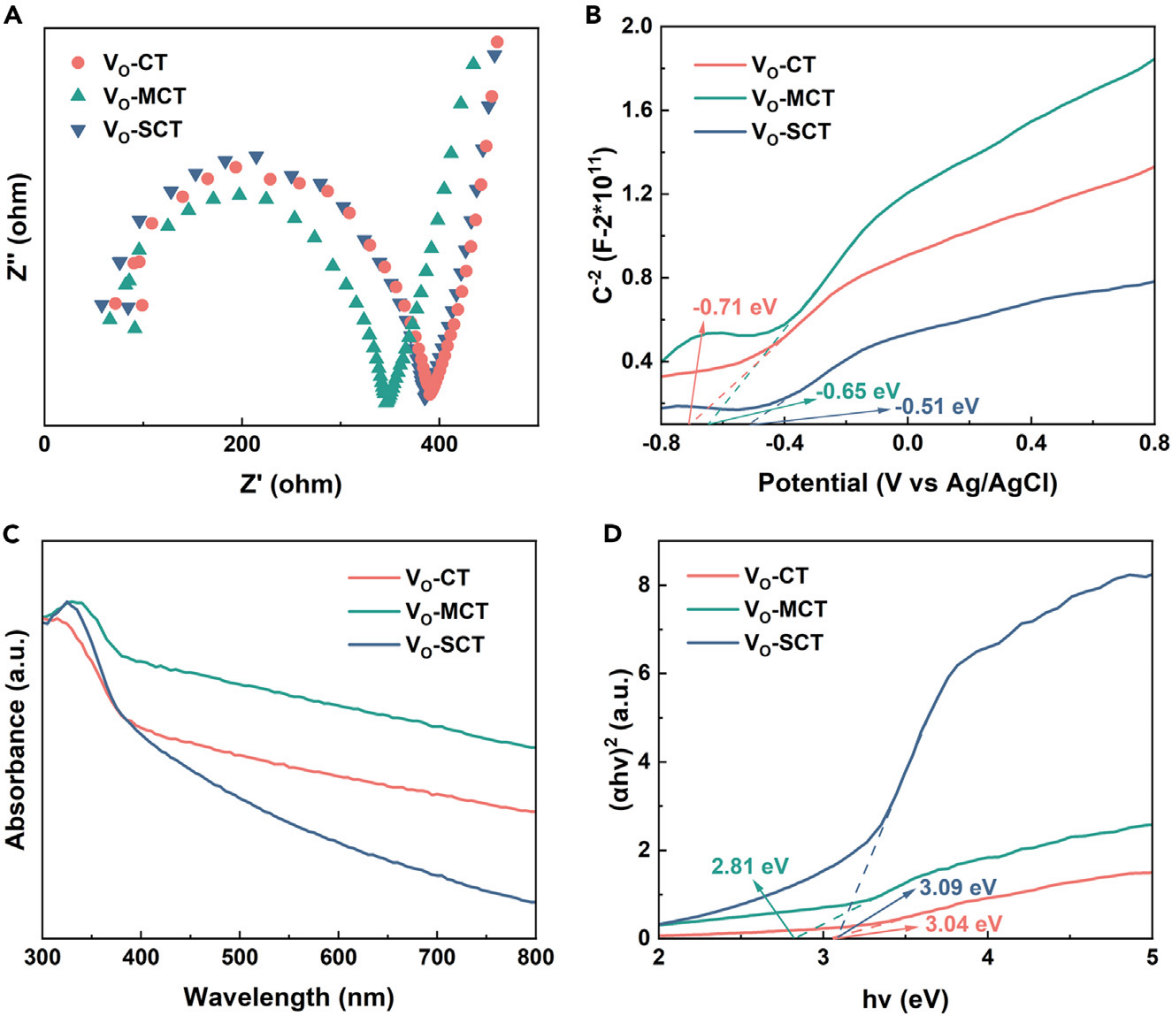
Characterization of photovoltaic properties of Vo-T, VO-MCT, and VO-SCT (A) Electrochemical impedance spectroscopy. (B) Mott-Schottky plots. (C) UV-visible absorption spectra. (D) Band gap energy diagrams. See also Figure S4.

From the EIS graph (Figure 6A), it can be observed that V_O_-MCT has the lowest charge transfer resistance, indicating the highest electron transfer rate. MS plots are utilized to determine the band positions of the samples. According to the MS plot (Figure 6B), the flat potential values of V_O_-CT, V_O_-MCT, and V_O_-SCT relative to the Ag/AgCl electrode are −0.71, −0.65, and −0.51 V, respectively. A lower flat potential value indicates better charge transfer.^31^^,^^32^ Although the flat band potential of V_O_-SCT is lower than that of V_O_-MCT, the specific surface area of V_O_-SCT is small, and a large number of oxygen vacancy defects cannot participate in surface reactions, resulting in low catalytic activity. Compared with V_O_-CT and V_O_-SCT, V_O_-MCT exhibits better absorption performance in the UV-visible region (Figure 6C). It may be due to the synergistic effect of oxygen vacancies and mesoporous structure, which enhances the light absorption ability of VO-MCT.^33^ Based on the Kubelka-Munk equation, the bandgap energy (Eg) of V_O_-CT, V_O_-MCT, and V_O_-SCT is calculated as 3.04, 2.81, and 3.09 eV, respectively (Figure 6D). A lower bandgap width indicates a lower energy required for electrons in the valence band (VB) to transition to the conduction band (CB). V_O_-MCT exhibits the lowest bandgap, reducing electron transfer energy and solving the problem of high bandgap in TiO_2_. The surface morphology and surface potential of the V_O_-MCT sample are characterized (Figure S4). Optoelectronic testing confirms V_O_-MCT has more superior performance, including faster charge carrier transport, more negative band edge positions, better light absorption, and lower bandgap.

### Photocatalytic CO_2_ reduction and mechanism

**Figure 7:**
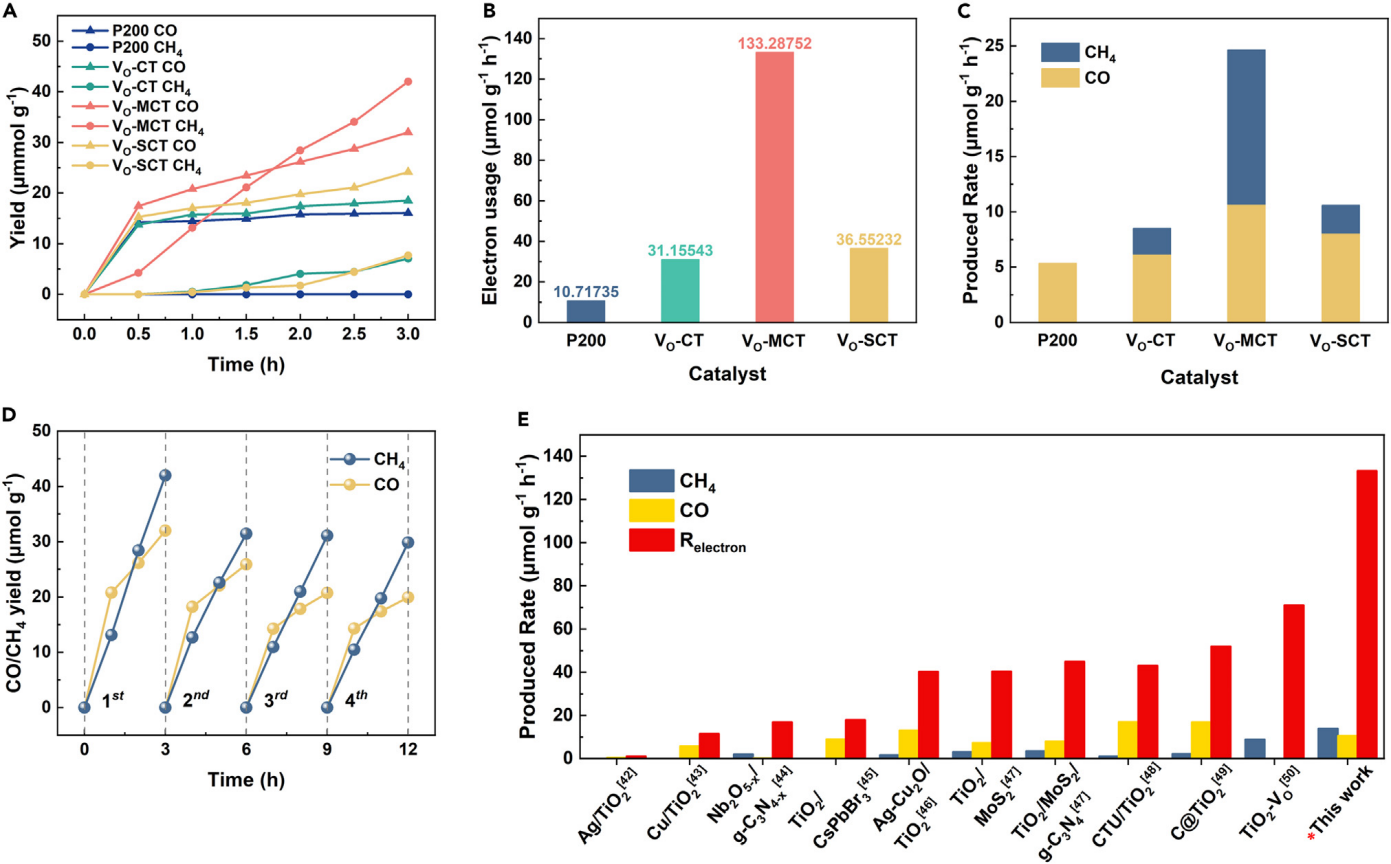
Photocatalytic CO_2_ reduction performance (A) CH_4_ and CO yields. (B) Electron consumption rate. (C) Average CH_4_ and CO yields. (D) Loop test of V_O_-MCT. (E) Comparison chart with the relevant literatures on photocatalytic CO_2_. See also Figure S5.

The photocatalytic CO_2_ reduction performance is characterized in deionized water systems without sacrificial pores and additives, as shown in Figure 7. In Figure 7A, it can be observed that the reduction products of commercial TiO_2_ (P200) are only CO with 16.08 μmol g^−1^. The CO yield of V_O_-SCT (24.69 μmol g^−1^) is slightly higher than that of V_O_-CT (18.75 μmol g^−1^). The CH_4_ yields of V_O_-SCT and V_O_-CT are basically similar, with average yields of 7.65 μmol g^−1^ and 7.05 μmol g^−1^, respectively. However, the CH_4_ and CO yields of V_O_-MCT are both the highest, at 42 μmol g^−1^ and 31.98 μmol g^−1^, respectively. The CO yield of V_O_-MCT is higher than that of the V_O_-SCT, V_O_-CT, and P200 samples. The CH_4_ yield of V_O_-MCT is 5.5 times and 5.9 times higher than that of the V_O_-SCT and V_O_-CT samples. Total photocatalytic activity is evaluated using the electron consumption rate, as shown in Figure 7B. V_O_-MCT demonstrats outstanding electron consumption rate of 133.29 μmol g^−1^ h^−1^, which is 12.43 times higher than that of the commercial TiO_2_. V_O_-MCT with oxygen vacancies and mesoporous structure has the highest CO_2_ conversion efficiency. Figure 7C shows the comparison of the production rates of photocatalytic CO_2_ products. V_O_-MCT has the highest CH_4_ and CO production rates of 14.00 μmol g^−1^ h^−1^ and 10.65 μmol g^−1^ h^−1^. There is no significant change in CH_4_ production in V_O_-MCT through four CO_2_ reduction cycle tests (Figure 7D). Furthermore, TEM characterization shows that the sample morphology remains unchanged after four cycles, indicating excellent stability of the catalyst (Figure S5). From Figure 7E, it can be found that the V_O_-MCT in this work shows a higher conversion capacity of CO_2_ into CO, CH_4_, and electron consumption rate as compared to other catalysts.^17^^,^^34^^,^^35^^,^^36^^,^^37^^,^^38^^,^^39^^,^^40^^,^^41^ The exceptional photocatalytic activity of V_O_-MCT can be attributed to the synergistic effect of oxygen vacancies and mesoporous structures. The oxygen vacancies act as an active site on the surface, promoting the adsorption of CO_2_ gas. Meantime, the mesoporous structure provides the catalyst with a larger surface area and higher pore structure, resulting in the formation of more electron-hole pairs, i.e., photocarrier generation. Thus, the V_O_-MCT with mesopores and oxygen vacancies exhibits better performance.

**Figure 8:**
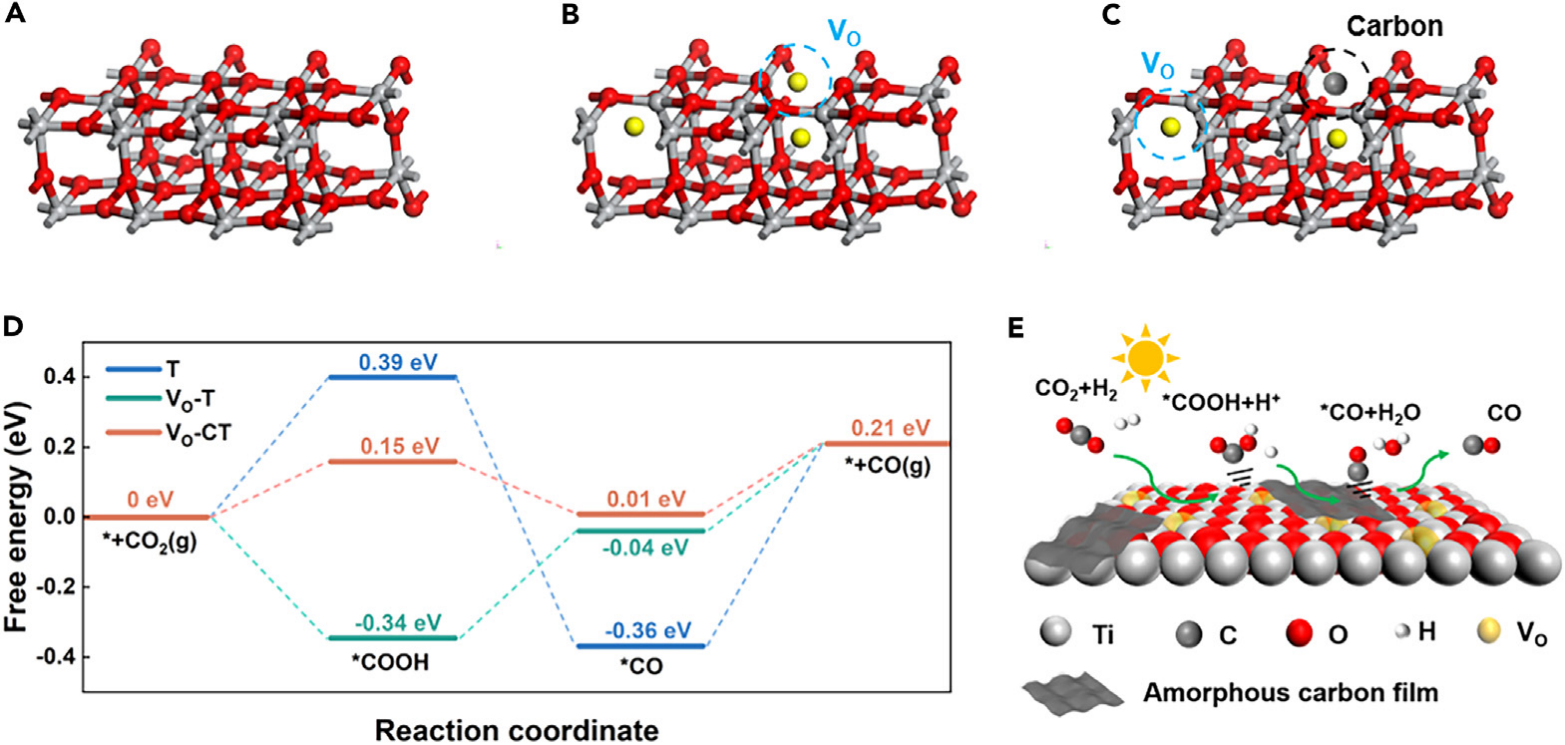
DFT calculations (A–C) The simulation structures of (A) T, (B) V_O_-T, (C) V_O_-CT. (D) Reaction pathways for CO_2_ reduction. (E) Reaction mechanism diagram of V_O_-CT in the simulated region. "∗" represents adsorption on the substrate. See also Figure S6 and Table S1.

To explain the CO_2_ reduction processes of three models, the Gibbs free energy (ΔG) of CO_2_ photoreduction is calculated, as shown in Figure 8D. In the intermediate state of ∗COOH adsorption, the ∗COOH molecules in both V_O_-T and V_O_-CT are closer to the position of oxygen vacancies than that of T, see Figure S6. It indicates a tighter interaction between the adsorbed molecule and the catalyst in V_O_-T and V_O_-CT. The presence of oxygen vacancies stabilizes the adsorption of CO_2_ molecules on the TiO_2_(101) surface.^42^^,^^43^ After CO_2_ adsorption on the T surface, it simultaneously absorbs an H^+^ and an e^−^ to form ∗COOH with ΔG = 0.39 eV. Then, ∗COOH continues to obtain e ^-^ and H^+^ to convert into ∗CO, with a negative Gibbs free energy, indicating a spontaneous reaction. Finally, ∗CO desorbs from the T surface to form CO with ΔG = 0.57 eV, which represents the maximum energy required in this reaction. For V_O_-T, the Gibbs free energies V_O_-T required for the transition from ∗COOH to ∗CO and from ∗CO to CO are 0.30 eV and 0.25 eV, respectively. Finally, ∗CO desorbs from the surface to form CO with ΔG = 0.25 eV. For V_O_-CT, the Gibbs free energies required for the transition from ∗CO_2_ to ∗COOH and from ∗CO to CO are 0.15 eV and 0.20 eV, respectively. Thus, the minimum energy required for the spontaneous CO_2_RR reaction of T, V_O_-T, and V_O_-CT are 0.57 eV, 0.30 eV, and 0.20 eV, respectively. Due to the combined effect of carbon and V_O_, V_O_-CT exhibits superior CO_2_RR activity theoretically. The calculated energy gaps, work functions, and Gibbs free energy of activation for the three models are presented in Table S1. The DFT simulation results demonstrate that oxygen vacancies are beneficial for the separation and transport of charge carriers, and the defect energy levels caused by oxygen vacancies are beneficial for electron capture. Meanwhile, the presence of C helps to reduce the energy barrier of the CO_2_ reaction.

### Conclusion

In summary, the synergistic effect of mesoporous structure and oxygen vacancy defect engineering is crucial for the photocatalytic CO_2_ reduction reaction of TiO_2_ materials. Density functional theory (DFT) calculations confirm that introducing oxygen vacancies and C atom on the TiO_2_ (101) surface effectively reduces the work function and creates intermediate defect energy levels, thus remarkably enhancing both electron transfer and electron capture capabilities. The presence of oxygen vacancies improves electron transfer rates, capturing more electrons for CO_2_ reduction reactions. Amorphous carbon films, acting as electron channels, not only assist in electron transport, but also contribute to the further reduction of energy bandgap and work function. In theory, the minimum energy required for the CO_2_RR reaction of TiO_2_ with oxygen vacancies and carbon atoms is reduced by 0.37 eV compared to pure TiO_2_. Through a simple sol-gel method and sintering method, P123 surfactant template and oxalic acid as inducer, carbon coated mesoporous TiO_2_ with oxygen vacancies (V_O_-MCT) is successfully synthesized at 450°C. The presence of oxygen vacancies and C is confirmed by XRD and XPS. When there are oxygen vacancies, electrons near Ti^4+^ ions can rearrange and form vacancy induced energy levels, thereby forming Ti^3+^. Ti^3+^ has partially filled d orbitals, which significantly alter the electronic structure and band structure of the material. TEM, SEM, and BET tests have confirmed the existence of mesoporous structures and hollow channels in Vo-MCT, with a specific surface area of 511 m^2^/g. The CH_4_ and CO yields of V_O_-MCT are both the highest, at 14.00 μmol g^−1^ h^−1^and 10.66 μmol g^−1^ h^−1^, respectively. The CO yield of V_O_-MCT is twice that of P200 samples. The CH_4_ yield of V_O_-MCT is 5.5 times and 5.9 times higher than that of the V_O_-SCT and V_O_-CT samples. Total photocatalytic activity is evaluated using the electron consumption rate, V_O_-MCT demonstrats outstanding electron consumption rate of 133.29 μmol g^−1^ h^−1^, which is 12.43 times higher than that of the commercial TiO_2_. Thus, V_O_-MCT demonstrates excellent photocatalytic activity in the CO_2_ reduction reaction, attributed to the synergistic effect of mesopores and oxygen vacancies. These results provide direction for the development of high-performance photocatalysts from both macroscopic structure and microscopic defects.

## STAR★Methods

### Key resources table

**Table:** 

REAGENT or RESOURCE	SOURCE	IDENTIFIER
**Chemicals, peptides, and recombinant proteins**		

Polyether P123	Macklin	9003-11-6
Tetrabutyl titanate	Macklin	5593-70-4
Potassium ferricyanide	Macklin	13746-66-2
Ethanol	Macklin	64-17-5
Oxalate	Aladdin	144-62-7
Glacial acetic acid	Macklin	64-19-7

### Resource availability

#### Lead contact

Further information and requests for resources should be directed to and will be fulfilled by the lead contact, Binxia Yuan (yuanbinxia100@163.com).

#### Materials availability

This study did not generate new materials.

#### Data and code availability

• Any additional information required to reanalyze the data reported in this paper is available from the lead contact upon request.
• Data Availability Statement: All data reported in this paper will be shared by the lead contact upon request.
• Code: This paper does not report original code.

### Method details

#### Materials

All reagents are of analytical-grade purity and used without further purification. Ti(OC(CH_3_)_3_)_4_ (TBOT), block copolymer surfactant (EO_20_-PO_70_-EO_20_, P123) with a molecular weight of 5800 g/mol, ethanol, and glacial acetic acid are purchased from Macklin. Oxalic acid is obtained from Aladdin. P200 (DHS-P200) for 200 nm-sized anatase crystalline TiO_2_ particles, is procured from Dalian HeptaChroma SolarTech Co., Ltd.

#### Preparation of Vo-T, VO-MCT, and VO-SCT

Firstly, 0.457g of P123 is dispersed in 30 mL of ethanol and stirred for 60 min. Subsequently, 1 mL (2.938 mmol) of TBOT and 0.7 mL (12 mmol) of glacial acetic acid are sequentially added, followed by different amounts of oxalic acid. The solution is placed in a drying box oven and polymerized at 40°C for 12 h, then dry overnight at 60°C. The resulting gel is transferred to a quartz crucible and annealed in a muffle furnace at 450°C for 4 h with a heating rate of 1 °C/min and a cooling rate of 2 °C/min. The samples obtained with different oxalic acid concentrations of 0 g, 0.3 g, and 2.1 g are named V_O_-CT, V_O_-MCT, and V_O_-SCT, respectively. V_O_ represents oxygen vacancy defects, C represents the encapsulation of amorphous carbon films, and M and S represent mesoporous and sealed mesoporous structures.

#### Characterization of the catalyst

X-ray diffraction (XRD) patterns are obtained using a Bruker D8 Advance X-ray diffractometer. EPR spectra are acquired using an ESR-5000 spectrometer. The oxygen vacancy concentration is determined by referencing the spin number of Cr(III) lone electrons in MgO, which is a stable and highly sensitive EPR material.^44^ The morphologies and microstructures are analyzed using a field-emission scanning electron microscope (SEM, FEI-400F) and transmission electron microscope (TEM, JEM-2100). The surface electronic states of the samples are analyzed via X-ray photoelectron spectroscopy (XPS, ESCALAB 250Xi), with all binding energies referenced to the C1s peak at 284.8 eV of the surface adventitious carbon. The Brunauer-Emmett-Teller (BET) specific surface area is estimated using a nitrogen adsorption-desorption analyzer (ASAP 2460, Micromeritics Inc.). UV-vis diffuse reflectance spectra (DRS) are obtained using a Shimadzu UV-3600 recording spectrophotometer.

#### Electrochemical measurement

The electrochemical measurements are conducted using the CHI760E electrochemical workstation. In a typical three-electrode setup, an Ag/AgCl electrode and Pt foil are used as the reference electrode and counter electrode, respectively. The working electrode is a sample film with dimensions of 1 × 1.5 cm^2^ prepared on FTO conductive glass. 2.5 M K[Fe(CN)_6_] electrolyte is used for measuring the electrochemical impedance spectroscopy (EIS) and Mott-Schottky (MS) curves. The illumination source is a 500 W xenon lamp with a full band spectrum.

#### Photocatalytic performance

The photocatalytic reduction of CO_2_ is carried out using a closed-loop gas circulation intelligent testing system provided by Beijing Magneson Co., Ltd. In this experiment, 50 mg catalyst is added to 100 mL of deionized water and stirred evenly. The Illumination source is a 300W xenon lamp (wavelength >300 nm, provided by Beijing Magneson Co., Ltd.). During illumination, the gas generated is intelligently collected and analyzed using a gas chromatograph (GC9790 II, R&F) equipped with a flame ionization detector (FID) and methanator. The total activity of the corresponding C product can be evaluated using the electron consumption rate (*R*_eletron_),^45^ which is calculated by the following Equation 1.

(Equation 1) $$R_{\text{eletron}}=R_{\text{CO}}\times K_{1}+R_{\text{CH}4}\times K_{2}$$

Where *R*_eletron_ is the electron consumption rate (μmol g^−1^ h^−1^), *R*_CO_ and *R*_CH4_ are the reaction rates of carbon monoxide and methane (μmol g^−1^ h^−1^), *K*_1_ is the number of electrons transferred for CO generation, and *K*_2_ is the number of electrons transferred for CH_4_ generation.^46^ Please refer to Equations 2 and 3 for further details.

(Equation 2) $${\text{CO}}_{2}+{2H}^{+}+{2e}^{-}\to \text{CO}+H_{2}O$$

(Equation 3) $${\text{CO}}_{2}+{8H}^{+}+{8e}^{-}\to {\text{CH}}_{4}+H_{2}O$$

#### DFT calculation models and method

All plane wave density functional theory (DFT) calculations are studied using the DMol3 package in Materials Studio software.^47^ It is calculated through PBE functional in Generalized Gradient Approximation (GGA).^48^ The (101) facet constructed by a 1 × 3 × 1 TiO_2_ supercell (72 atoms) is used for modeling with the thickness of the vacuum layer to 15 Å, named T. The convergence accuracy of the self consistent field (SCF) is set to Coarse (1 × 10^−4^), the base set is set to DN, the maximum self consistent cycle is set to 100, the Use smoothing is checked and set to 0.05Ha, the orbital truncation energy is set to Custom grid parameters, and the k-point is set to 2 × 2 × 1. Three oxygen atoms are removed from the optimized TiO_2_ structure, named V_O_-T. A C atom is added on this basis to simulate carbon overlay, named V_O_-CT. The above three models are shown in Figure S1. After optimization, its energy band, density of states, work function, and other properties are calculated. Then, adsorb ∗COOH and ∗CO as intermediate states on the optimized structure, and select them as HessianAtoms to calculate their frequency. Convergence with the force on each atom is set below 0.02 Ha/Å, the energy on each atom is within 1 × 10^−4^ eV and the displacement on each atom is within 0.05 Å for all calculations. Adsorption energy is calculated as the following Equation 4:

(Equation 4) $${\Delta E}_{\text{abs}}=E_{\left(\text{slab}+\text{absorbate}\right)}-E_{\text{slab}}-E_{\text{absorbate}}$$

The *E*_slab_ and *E*_absorbate_ represent the energies of the two isolated subsystems, respectively. *E*_(slab+absorbate)_ is total energy of the interacting slab-adsorbate system. The charge density difference is calculated as the following Equation 5:

(Equation 5) $$\Delta \rho =\rho_{\left(\text{slab}+\text{absorbate}\right)}-\rho_{\text{slab}}-\rho_{\text{absorbate}}$$

Where *ρ*_slab_ and *ρ*_absorbate_ stand for the charge densities of the two noninteracting subsystems separately, whereas *ρ*_(slab+absorbate)_ means the charge density of the interacting slab-adsorbate system.

The reaction free energy changes are calculated as following Equation 6:

(Equation 6) $$\Delta G=\Delta E-{\Delta E}_{ZPE}-T\Delta S$$

The Δ*E*, Δ*E*_*ZPE*_, and Δ*S* are electronic energy, zero-point energy, and entropy difference between products and reactants, respectively. The vibrational frequencies and entropies of molecules in the gas phase are taken from the NIST database.^49^
