# Supplementary material for: Synergistic effects of oxygen vacancies and mesoporous structures in amorphous C@TiO2 for photocatalytic CO2 reduction
Source: iScience. 2024 Jun 25;27(7):110377. doi: 10.1016/j.isci.2024.110377 (PMC11269941; doi:10.1016/j.isci.2024.110377)
Supplement: Document S1. Figures S1–S6 and Table S1 [file mmc1.pdf]

iScience, Volume *27*

Supplemental information

Synergistic effects of oxygen vacancies and mesoporous structures in amorphous $C@TiO_2$ for photocatalytic CO_2 reduction

Binxia Yuan, Yuhao Liu, Hong Qian, Rui Zhu, Chengxi Zhang, and Weiling Luan

Supporting information

Fig. S1A illustrates the constructed atomic model of T. Fig. S1B shows V_O-T model obtained by removing three oxygen atoms from the corresponding positions in T model. The yellow oxygen atom within the blue dashed circle represents the oxygen vacancy. V_O-CT is obtained by placing one carbon atom on top of one of the oxygen vacancies in V_O-T to simulate the encapsulation of an amorphous carbon film (Fig. S1C).

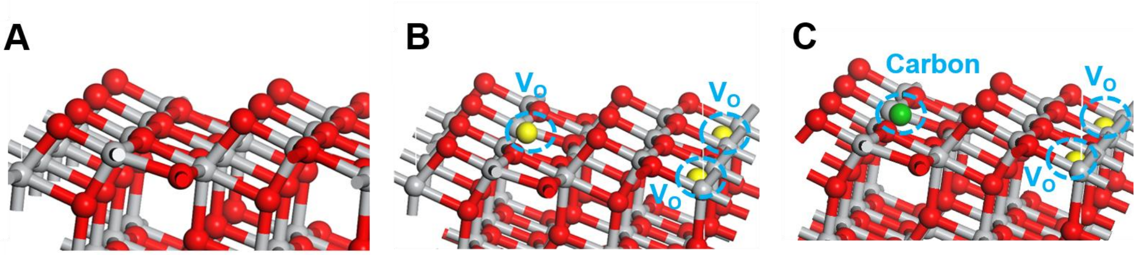

Fig. S1. Atomistic model of exposing (101) surface, related to Fig. 1.

(A) T.

(B) VO-T.

(C) VO-CT.

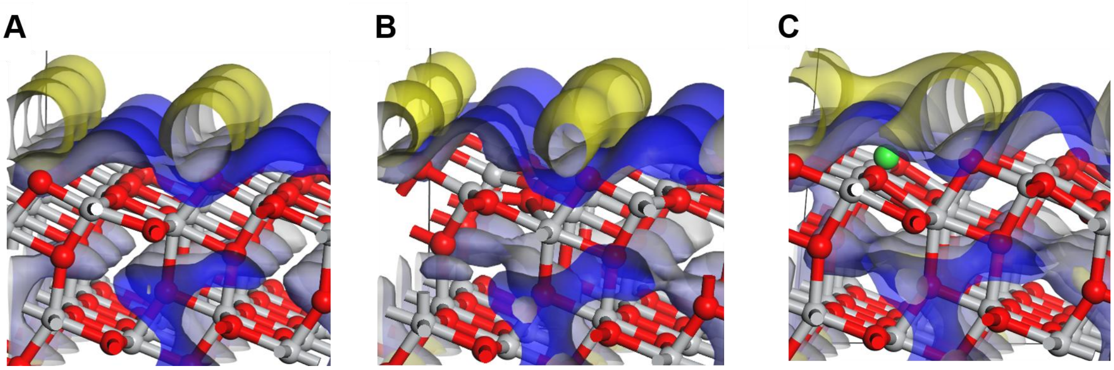

Fig. S2. Differential charge density maps, related to Fig. 1.

(A) T.

(B) V_O-T.

(C) V_O-CT.

The effects of varying annealing temperature and surfactant P123 content on the structural characteristics of the sample are investigated using SEM observations in Fig. S3. The annealing temperature increases from 450°C to 650°C, and the sample is composed of uniform small particles (Fig. S3B). The variables of P123 are changed to 0.1g and 1g respectively (Fig. S3C and S3D), and the sample morphologies are similar and composed of small particles. The samples are designated as P123-0.3-650, 0.1-P123-0.3, and 1-P123-0.3.

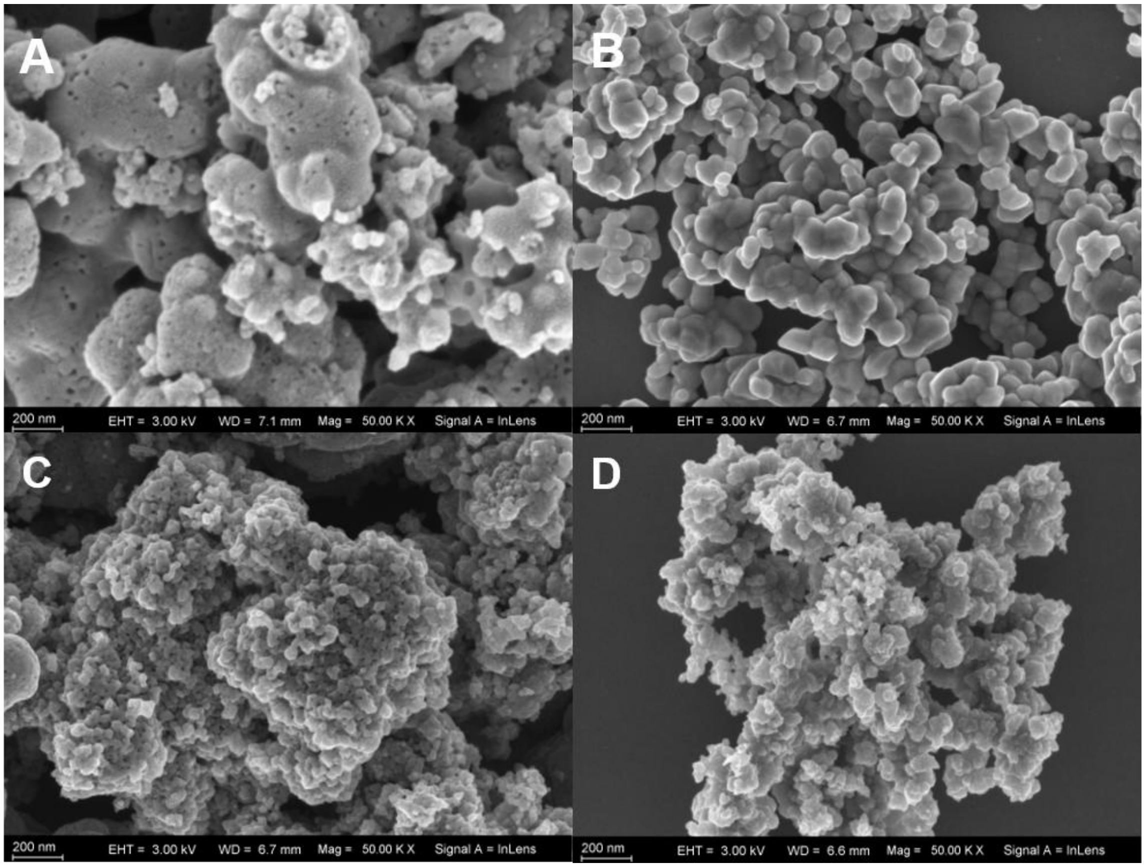

Fig. S3. SEM image, related to Fig. 4.

(A)V_O-MCT.

(B)P123-0.3-650℃

(C)0.1-P123-0.3.

(D)1-P123-0.3.

Through scanning probe microscopy (SPM), the surface morphology and surface potential of the V_O-MCT sample are observed. In Fig. S4A, it can be seen that V_O-MCT exhibits a rough surface with a high specific surface area. However, SPM can only scan the external morphology and cannot observe the internal pore and hollow structures of the sample like a transmission electron microscope (TEM). The observed depressions in the image may be attributed to the surface mesoporous structure. In Fig. S4B, a remarkably uniform surface potential is observed on the V_O-MCT sample, indicating a homogeneous distribution of surface charges.

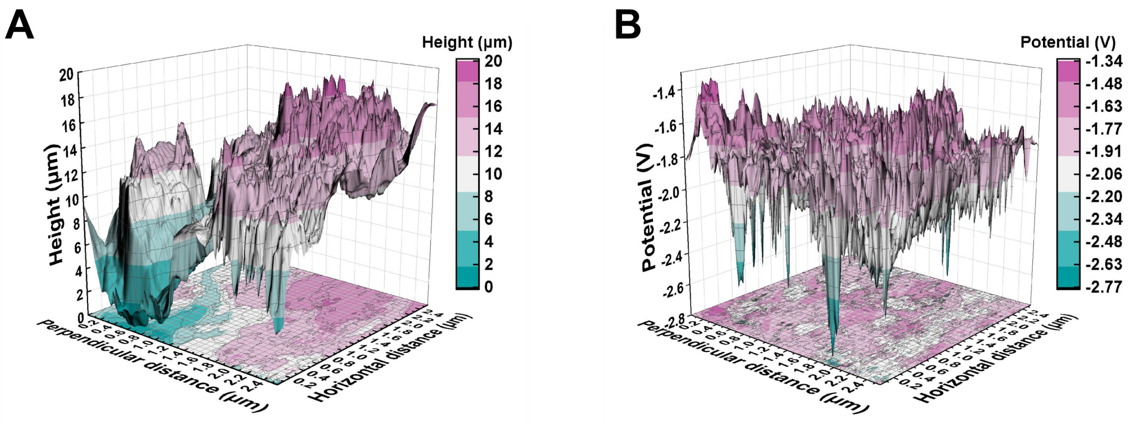

Fig. S4. SPM images of the VO-MCT, related to Fig. 6.

(A) Surface Topography.

(B) Surface Potential.

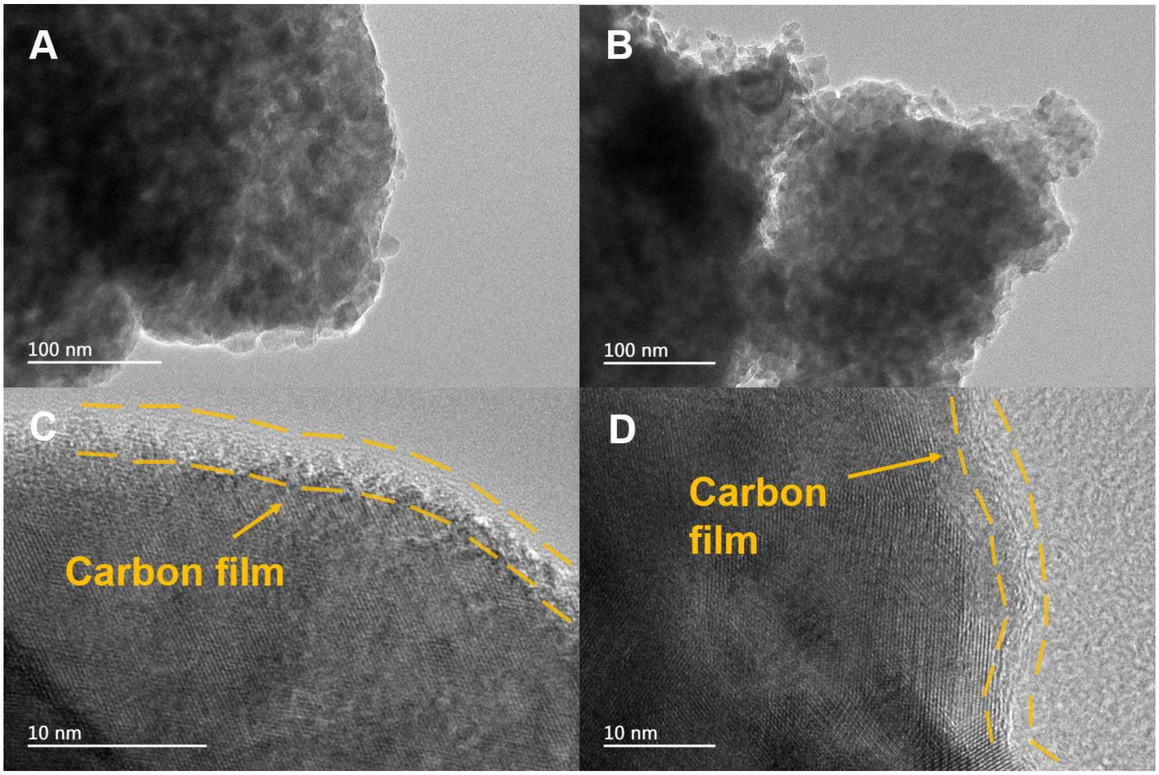

Fig. S5. The TEM and HRTEM images of the V_O-MCT sample, related to Fig. 7.

(A,C) before the photocatalytic CO_2 reduction reaction.

(B, D) after four cycles of testing.

Compared to T surface adsorbing -COOH molecules, both V_O-T and V_O-CT exhibit a more compact configuration and tighter interaction with the material surface after optimization (Fig. S6).

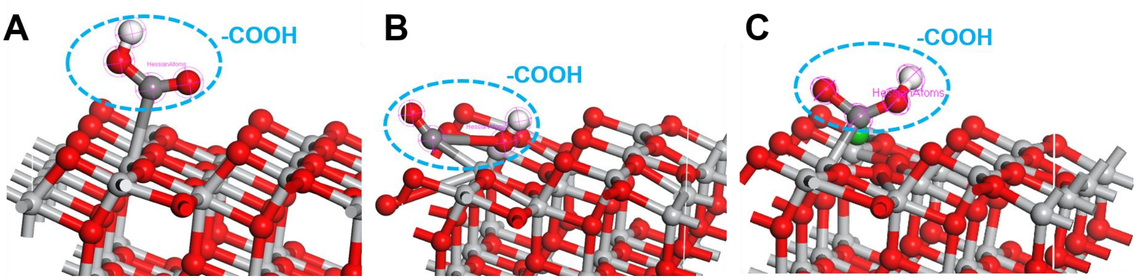

Fig. S6. Intermediate state atomistic model, related to Fig. 8.

(A) T surface adsorbing -COOH.

(B) V_O-T adsorbing -COOH.

(C) V_O-CT adsorbing -COOH.

Table S1. Energy bandgaps and work functions of T, V_O-T, and V_O-CT, related to Fig. 8.

Model	Energy gap (eV)	Work Function (Ha)	ΔG_{max} (eV)
T	3.684	0.280	0.57
V_O-T	2.231	0.265	0.30
V_O-CT	1.371	0.227	0.20
